# Dreaming

**Published:** 1917-01

**Authors:** 


### Dreaming.

If we were in the habit of dreaming as many apparently normal persons are, we should dislike to fall into the hands of the modern scientific dream experts. The analysis of dreams runs somewhat as follows: One dreams of visiting a farm. Farmer, in German, is Bauer, and the same word is used for the Jack in cards, indeed the same word, spelled phonetically, is also used in English. Hence, to dream of visiting a farm indicates that the dreamer is a gambler, or would like to be if he had the necessary skill. In a rather limited personal experience, it has seemed to us that dreams depended mainly on external stimuli or recollections of non-personal occurrences, and not to a great degree on the subjective element, including the workings of the sub-conscious ego.

This subconscious self is another matter that bothers us. We would like to see the next article on the subject preceded by a demonstration of a cortical center or, at least, connected with the older spiritual psychology. There are functions controlled by centers not in any way connected with the ordinary intellectual ones and others, originally voluntary, that become more or less automatic, it would be foolish to deny. Nor can we deny that we often think "absent-mindedly" or show peculiar perversions of intelligence under anaesthesia, intoxication in either the narrow or the broad sense, or when fully asleep or drowsy. What we object to, is the use of the term subconscious self, as if each individual were a twin, and as if this self were a demonstrated psychologic and physiologic entity.

Having several times gone over examinations for library assistants and marked ourself 15-20%, and having been often puzzled by the expressions used by small children and very illiterate persons, and obviously conveying to them a very simple meaning, we would dislike to try a mental test according to the modern system. We have visions of being catalogued as mentally developed to the stage of 11 years, 3 months and 18 days, and of being sent to an institution.

We are striving to lead an exemplary life and to avoid anything that would place us under suspicion of having committed a crime. Imagine the inquisition by a devotee of the newer psychology of association. For example, if it were charged that we had drowned someone, we should be so afraid that some one of the list of words fired at us would suggest water that we would not even dare mention ice or hydrogen, and our endeavor to avoid even dampness would certainly lead to a few seconds hesitation on some innocent word or to a too rapid and not sufficiently obvious suggestion of something kiln-dried. And then it would be only a few simple steps to the 20,000 volt current, for in such a test one must be neither too rapid nor too slow and, unfortunately, we are one of several million whose brains do not always work at the same speed.

There are other modern developments, but we shall close with a toast to the Unimaginative, Pragmatic Man.

